# Generalized Aggressive Periodontitis and Its Treatment Options: Case Reports and Review of the Literature

**DOI:** 10.1155/2012/535321

**Published:** 2012-01-12

**Authors:** T. Roshna, K. Nandakumar

**Affiliations:** ^1^Department of Periodontics, People's Dental Academy, Bhopal 462010, India; ^2^Department of Periodontics, Azeezia Dental College, Kollam 691537, India

## Abstract

Generalized aggressive periodontitis results in rapid destruction of the periodontium and can lead to early tooth loss in the affected individuals if not diagnosed early and treated appropriately. The diagnostic features of the disease are characteristic, but the clinical presentation and patterns of destructions may vary between patients. Successful management of the disease is challenging especially if diagnosed at advanced stages of the disease, but not impossible with the current therapeutic choices for the disease. A vast array of treatment modalities is available which can be employed in the treatment of generalized aggressive periodontitis with varying success rates, but a definite guideline for the management is yet to be formulated. However, with the exponential rate of developments in periodontal research, regenerative therapy, tissue engineering, and genetic technologies, the future seems promising in regard to options at managing the disease. This paper attempts to describe the clinical and radiographic diagnostic features and the current treatment options along with a suggested protocol for comprehensive management of generalized aggressive periodontitis patients with case reports and a brief review.

## 1. Introduction

Aggressive periodontitis, as the name implies is a type of periodontitis where there is rapid destruction of periodontal ligament and alveolar bone which occurs in otherwise systemically healthy individuals generally of a younger age group but patients may be older [1, 2]. Although its prevalence has been reported to be much less than that of chronic periodontitis, it can result in early tooth loss in the affected individuals if not diagnosed in the early stages and treated appropriately [3]. The disease is generally found to have a racial and sex predilection, with blacks and male teenagers having higher risk for the disease compared to whites and females, although reports vary between different ethnic groups and populations, with some populations showing prevalence as high as 28.8% [4, 5].

Aggressive periodontitis, first described in 1923 as “diffuse atrophy of the alveolar bone” [6], has undergone a series of terminology changes over the years to be finally named as “aggressive periodontitis” in 1999 [1, 7]. The disease which includes both localized and generalized forms was previously known as “early onset periodontitis” which included the three categories of periodontitis—prepubertal, juvenile, and rapidly progressing periodontitis [8, 9]. It is interesting that the first ever reported detailed description of a recognized disease in early hominid evolution is a case of prepubertal periodontitis in an 2.5–3-million-year-old fossil remains of a juvenile *Australopithecus africanus *specimen which showed the typical pattern of alveolar bone destruction with migration of the affected deciduous molars [10, 11].

Generalized aggressive periodontitis (GAgP) is characterized by “generalized interproximal attachment loss affecting at least 3 permanent teeth other than first molars and incisors” [12]. It is a multifactorial disease where interplay of microbiologic, genetic, immunologic, and environmental/behavioral risk factors decides the onset, course, and severity. Pathogenic bacteria in the dental plaque especially *Aggregatibacter actinomycetemcomitans and Porphyromonas gingivalis* [13, 14] have an indispensable role which elicits an aggravated host response which in turn is determined by the genetic and immunologic profile of the patient modified by environmental risk factors like smoking.

This paper attempts to describe the diagnostic features along with the periodontal management options of generalized aggressive periodontitis with the help of case reports with different clinical presentation and patterns of involvement and managed with different treatment modalities available. Finally an attempt to summarize the available protocol for a comprehensive management of GAgP is done which can serve as a guideline till more definite clear-cut guidelines are established for the disease in the future.

## 2. Clinical Features

**Figure 1:**
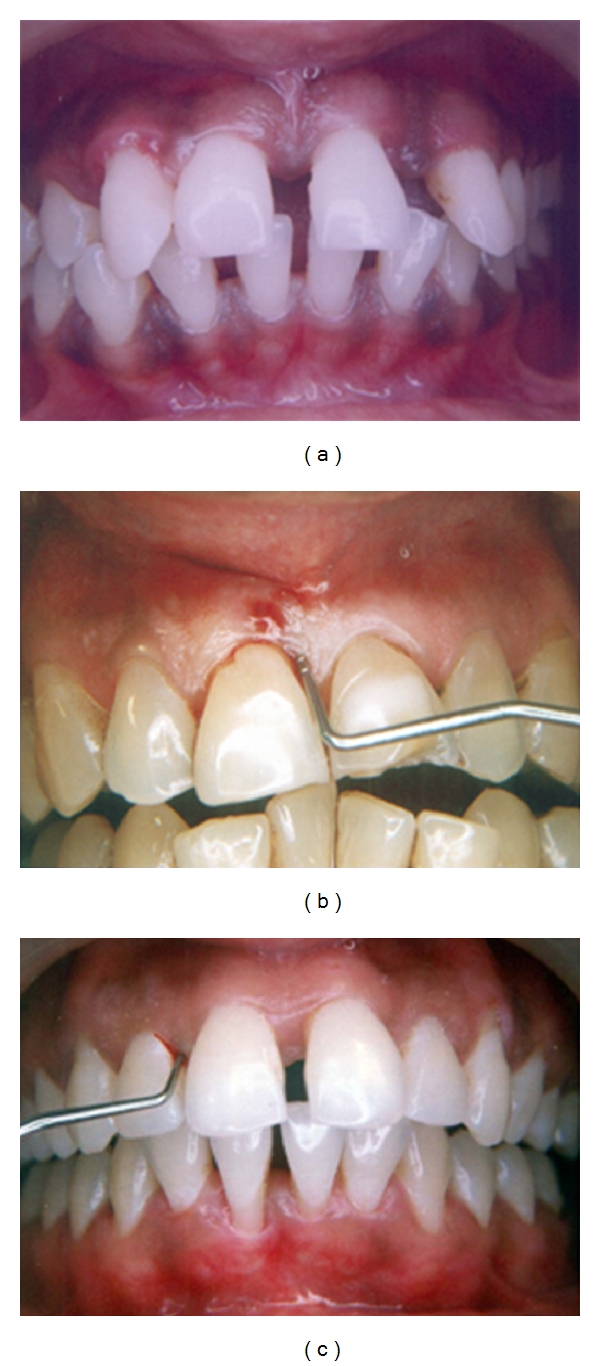
(a) Patient presenting with flaring of the anterior teeth. (b) and (c) Deep pockets revealed by probing in a periodontium with lack of clinical inflammation.

The most common reported complaints are a recently noticed flaring and progressing spacing of anterior teeth and bleeding from gums comparatively in a young patient but patients can be older as well (Figures 1(a)–1(c)).

Patients may complain of halitosis and pus discharge from gums. Mobility of the affected teeth will be seen towards the later stages of the infection. Patients will be otherwise systemically healthy. Severe pain is rarely experienced by the patients except in situations where a periodontal abscess develops or a periodontal-endodontic infection occurs via accessory canals or tooth apex. Some patients may complain of a dull nagging type of pain from gums. Gingival recession may be seen and patients may complain of food impaction due to loss of contact points between teeth.

**Figure 2:**
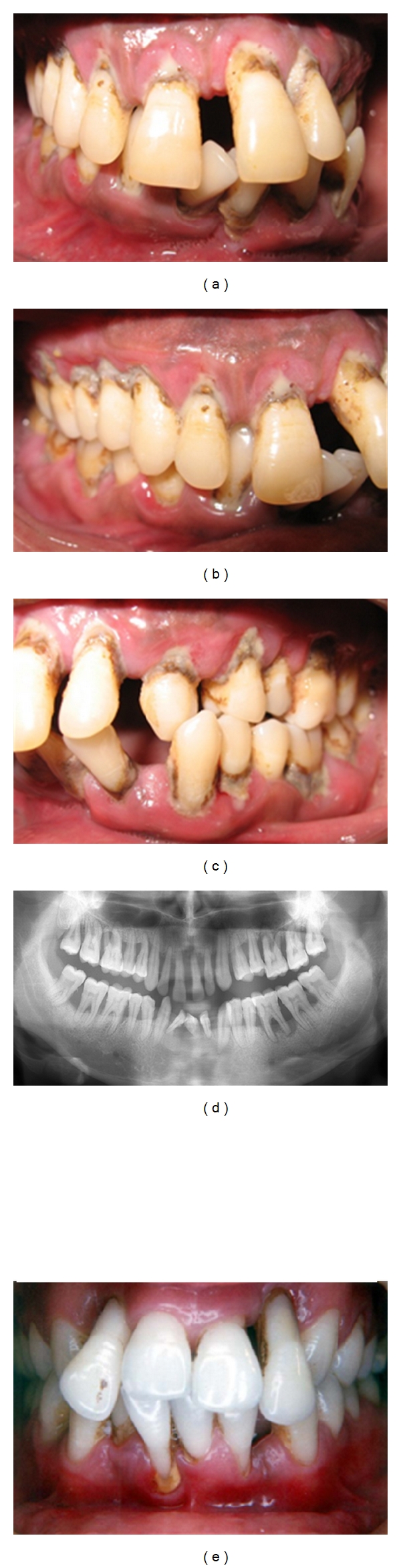
(a)–(d) Clinical and radiographic appearance of GAgP in a 25-year-old smoker with poor oral hygiene. (e) A 19-year-old smoker with GAgP demonstrating severe periodontal destruction.

GAgP patients who smoke and/or maintain a poor oral hygiene demonstrate more severe destruction of periodontium compared to those who do not smoke or maintain a satisfactory oral hygiene (Figures 2(a)–2(e)).

The disease progresses in alternating periods of activity and quiescence [15]. This leads to two types of presentation at the time of examination. In the periods of quiescence, patients are free of symptoms and the gingiva appears pink and healthy even though probing reveals deep periodontal pockets. Lack of visible signs of clinical inflammation despite the presence of deep periodontal pockets and severe attachment loss in an otherwise healthy young individual is the classic sign of aggressive periodontitis presenting at this stage (Figures 1(a)–1(c)). Probing should be done with calibrated periodontal probes at six sites around each tooth.

**Figure 3:**
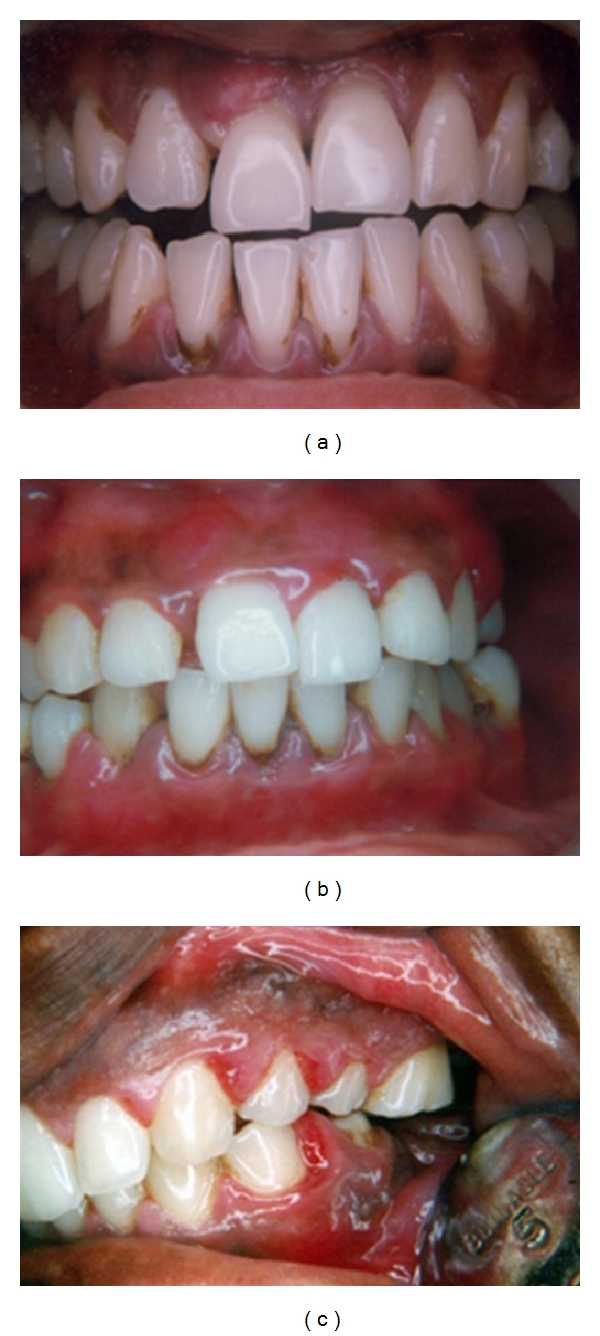
(a)–(c) Features of severe gingival inflammation presented at the stage of active disease.

The periods of inactivity may remain for weeks to months or even years and will be followed by periods of active disease. During this period, there will be active bone destruction and attachment loss. When the patient presents in this stage, the gingiva will show all signs of mild to severe inflammation. Gingiva may be tender, fiery red, edematous, soft, and boggy. Bleeding on probing or even spontaneous bleeding and purulent exudation may be evident. Inflammatory gingival enlargement may also be noticed. The majority of the patients refer to dental consultation at this stage of the disease (Figures 3(a)–3(c)).

 This stage may undergo spontaneous remission after a varying period of destruction and the inflammatory symptoms subside to reappear after a period of quiescence. Advanced stages of the untreated disease with severe periodontal destruction may show extrusion of teeth, mobility and pathologic migration, furcation involvement, generalized gingival recession, and loss of several teeth due to spontaneous exfoliation. Some patients may show systemic manifestations such as weight loss, mental depression and general malaise [16].

## 3. Radiographic Features

**Figure 4:**
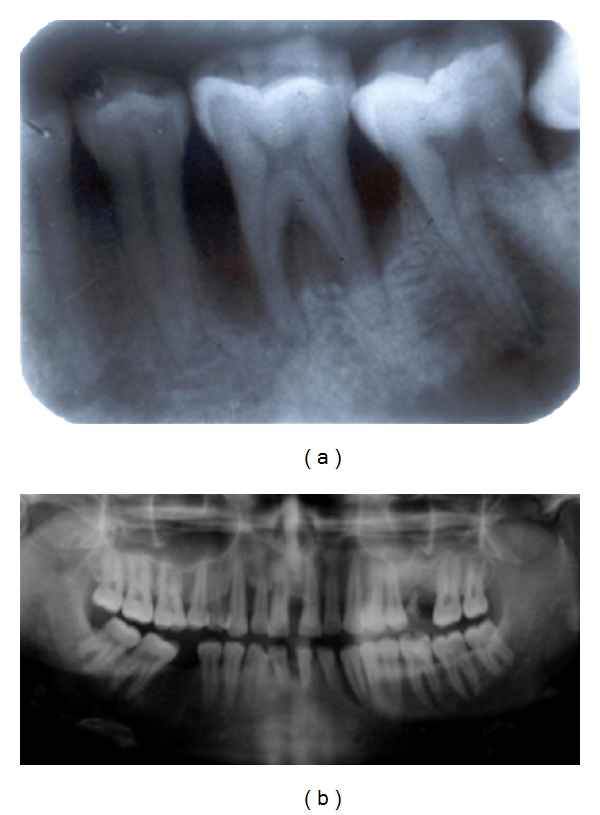
(a) Arc-shaped radiolucency at the 1st molar region in localized aggressive periodontitis. (b) Generalized distribution of bone loss seen in GAgP with vertical defects.

Localized aggressive periodontitis typically presents “arc-shaped” mirror image radiolucency in the first molars starting from the distal aspect of second premolars to the mesial aspect of the second molar. In generalized aggressive periodontitis, radiographs may show generalized bone destruction ranging from mild crestal bone resorption to severe extensive alveolar bone destruction depending on the severity of the disease. The defects may be a combination of vertical and horizontal defects (Figures 4(a) and 4(b)).

## 4. Diagnosis

Early diagnosis is of utmost importance for the prevention of extensive attachment loss and bone loss experienced in aggressive periodontitis. Diagnosis is made according to the criteria set by the American Academy of periodontology, 1999 classification of periodontal diseases and conditions [1], using history, clinical features, and radiographic features aided by microbial examination if needed. Family history may reveal a history of early tooth loss in the parents or immediate blood relatives of the patient [17]. The amount of microbial deposits will be inconsistent with the amount of destruction when compared to chronic periodontitis and plaque will be minimal. Comparison of serial radiographs helps in assessing the rapid rate of bone destruction and can aid in the diagnosis of the disease.

## 5. Differential Diagnosis

Aggressive periodontitis can be differentiated from chronic periodontitis by the age of onset, rapid rate of disease progression, the nature and composition of the associated subgingival microflora, alterations in host immune response, and a familial aggregation of the diseased individuals [18]. Systemic diseases like hematologic disorders and some genetic disorders also show periodontitis as a manifestation mimicking generalized aggressive periodontitis which can be ruled out by assessing the systemic status, hematologic data analysis, and immunologic profiling of the patient. In addition, there are rare reports of certain conditions like intraosseous sarcoidosis [19], eosinophilic granuloma [20, 21] and alveolar bone actinomycosis [22], presenting with extensive alveolar bone destruction like in aggressive periodontitis which can be differentiated by biopsy of the suspected lesions.

## 6. Case Reports

### 6.1. Case Report 1

**Figure 5:**
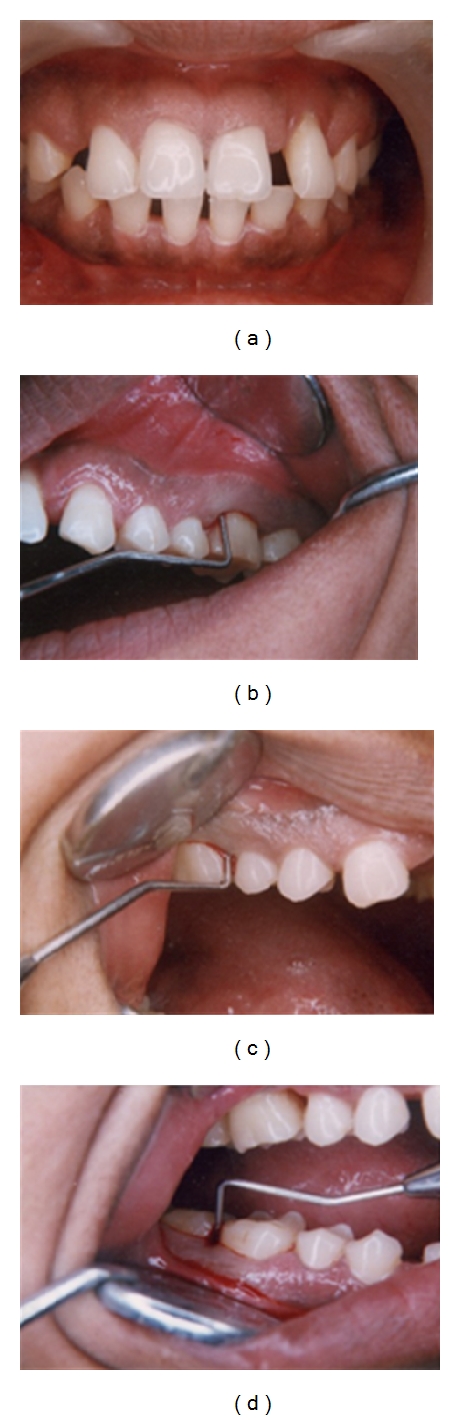
(a)–(d) Deep pockets were present even though signs of gingival inflammation other than bleeding on probing were absent.

A 32-year-old female patient presented with the complaint of a recently noticed spacing between the upper front teeth. (Figures 5(a)–5(d)).

The patient noticed the spacing about 1 year before, after which she noticed it to be gradually increasing and associated with intermittent episodes of pus discharge which subsided on taking antibiotics as per advice at a local hospital. There were no associated complaints other than a cosmetic concern from the patient. There was no history of any previous dental treatment. Family history of similar complaints or early tooth loss could not be elicited. The patient was systemically healthy with no relevant medical history.

There were no abnormalities detected in extra oral examination except for a slightly tender and palpable left submandibular lymph node. Full complement of teeth was present. The oral hygiene status of the patient was good as revealed by the oral hygiene index. There was minimal amount of calculus and plaque. There was grade I mobility of 22, 31, 32, 21 and 22. Proximal contacts were lost between the teeth 14 and 13, 13 and 12, 21 and 22 and 22 and 23, 22 and 24 and between lower anterior teeth. There was labial migration and flaring of upper and lower anterior teeth with an evident distolabial migration of 22.

Gingival examination revealed normal color except for the labial aspect of 22 where it was slightly reddish. The margins were of knife-edge contour except for the labial aspect of 22 and 42 where it was bluntly rounded. The gingiva was firm and resilient except in the region on 22 where it was soft in consistency. There was no loss of stippling in the anterior regions. The position of the gingival margin was apical to the CEJ in the labial aspect of 22. There was generalized bleeding on probing, and exudation was present on the labial aspect of 22. All together there were minimal signs of inflammation other than bleeding on probing.

**Figure 6:**
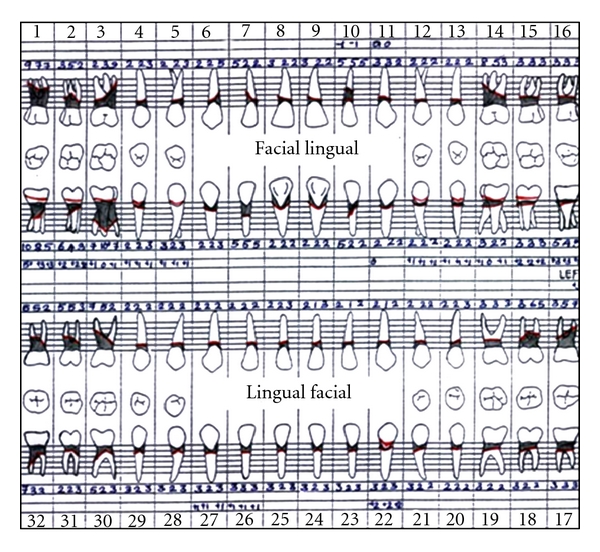
Periodontal charting showing generalized deep pockets and clinical attachment loss.

A full-mouth periodontal charting revealed generalized periodontal pockets and clinical attachment loss (Figure 6).

Pockets were especially deeper in the molar and incisor regions with slightly lesser involvement in the premolar region. The clinical attachment loss ranged from a maximum of 10 mm in the midpalatal aspect of 16 to a minimum of 2 mm in the premolar regions.

**Figure 7:**
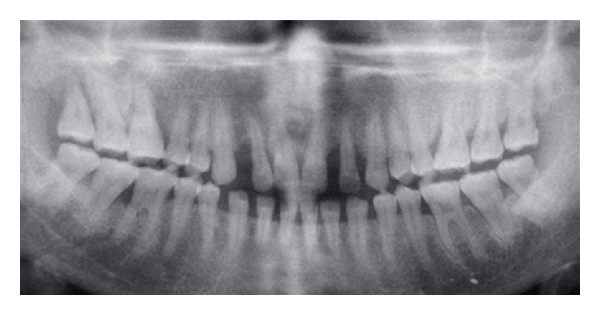
Orthopantomogram of the patient demonstrating generalized distribution of bone loss.

 An OPG and full-mouth IOPA X-ray were performed which revealed the generalized distribution of alveolar bone loss which was a combination of both horizontal and vertical bone loss (Figure 7). Routine blood examination results were within normal limits.

Based on the history, examination findings, and the radiographic findings, a diagnosis of generalized aggressive periodontitis was made according to the criteria by AAP 1999 classification.

### 6.2. Management

A thorough supragingival scaling was performed following which the patient was motivated for better plaque control. A sulcus brushing technique (modified Bass technique) [23] was demonstrated, and the patient was educated on the use of interdental cleansing aids including dental floss and interdental brushes. Chlorhexidine mouth wash was prescribed to further aid in plaque control. Systemic antibiotics (Amoxycillin and Metronidazole, 250 mg of each thrice daily) were prescribed for 8 days, and the patient was recalled after 2 weeks for evaluation of the response to treatment [24].

 A subgingival scaling was performed after which the patient was advised to continue the chlorhexidine mouthwashes. A reevaluation 2 weeks after subgingival scaling showed a reduction in probing depths and absence of bleeding on probing.

**Figure 8:**
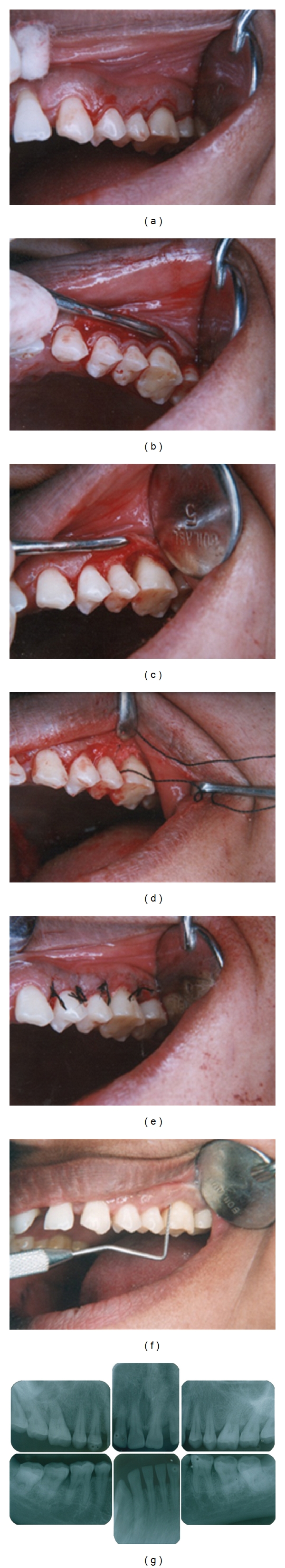
(a)–(e) Modified Widman flap in conjunction with bone grafting performed. (f) Postoperative evaluation showing probing depth within normal limits. (g) 6-Month postoperative intraoral periapical radiographs.

 A quadrant-wise full-mouth flap surgery was performed including bone grafting in relation to the molar regions where predominantly vertical or intrabony defects were detected. A modified Widman flap surgery [25] in conjunction with bone replacement graft was performed in the molar regions (Figures 8(a)–8(e)) whereas a sulcular incision flap (Kirkland flap) was performed in the maxillary and mandibular anterior region to minimize the recession after healing for esthetic purposes.

A preprocedural rinse with antimicrobial agent was done to minimize the bacterial count in the mouth. After adequately anesthetizing the surgical site with infiltration anesthesia and nerve blocks, the first incision (internal bevel incision) 0.5 mm from the gingival margin directing to the crest of the alveolar bone was made. The flap was reflected following which sulcular incision and interdental incision were made to remove the wedge of tissue. Curettage for granulation tissue removal was done following which a through subgingival debridement and root planning was performed. The defect was irrigated with normal saline, and a root conditioning with tetracycline was performed. The graft was a xenograft (Bovine graft—Ossopan), which was mixed with the blood from the surgical site and placed into the defect after presuturing the site with silk sutures. Care was taken to fill the graft to a realistic level and not to overpack the defect. Suturing was done after adapting the buccal and lingual flaps well. A periodontal pack was placed, and antibiotics and analgesics were prescribed for the patient for 5 days. A fluoride-containing mouthwash was prescribed postsurgically to the patient.

Healing was uneventful, and a postoperative evaluation 3 weeks after surgery showed absence of bleeding on probing and probing depths within normal limits (Figure 8(f)). The patient was put on regular recall appointments for evaluation of the gingival and periodontal status and maintenance therapy. A postoperative radiograph 6 months later showed a significant bone fill in the molar regions where grafting was done with an increase in bone density of the alveolar crest with corticated bone formations in the crest at the other areas (Figure 8(g)). The oral hygiene maintenance and compliance of the patient was excellent, and there were no signs of recurrence of the disease throughout the maintenance period. Since the patient was concerned about the esthetic appearance of the anterior teeth, she was advised to undergo adult orthodontic therapy after 1 year of surgery under regular periodontal monitoring and was referred to an orthodontic specialist for the same.

### 6.3. Case Report 2

**Figure 9:**
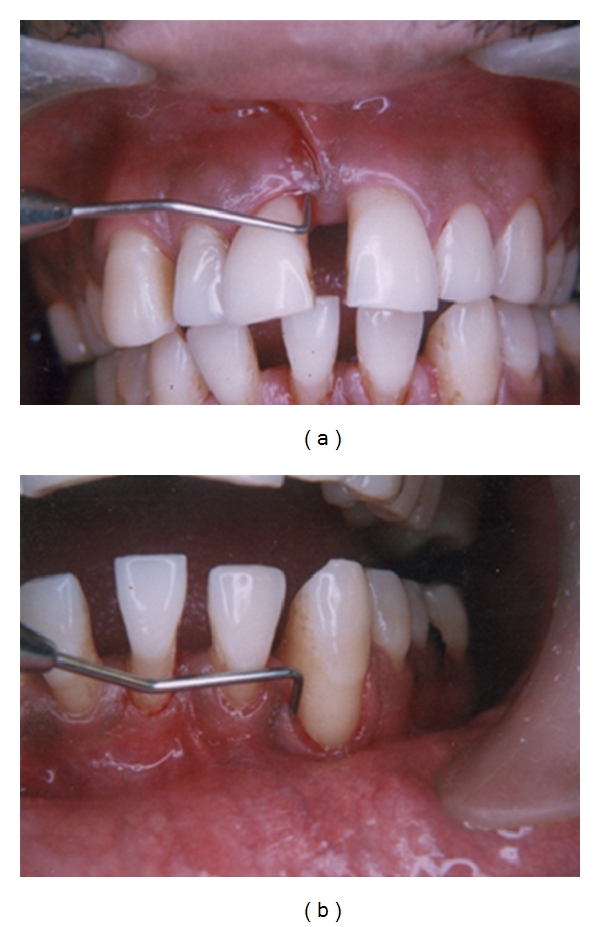
(a) and (b) Clinical presentation with deep pockets, recession, and pathologic migration of teeth.

A 26-year-old male patient presented with the chief complaint of generalized pus discharge from gums which he had been experiencing intermittently for the past 2 years (Figures 9(a) and 9(b)). Pus discharge was associated with bad breath and usually subsided spontaneously after a few weeks. There was no associated complaint other than a generalized mild hypersensitivity to cold and sweet food. He had a history of extraction of lower left posterior tooth due to caries exposure and extraction of lower front tooth due to mobility about 1 year before. There was no history of any other dental treatment.

The patient was systemically healthy, and medical history did not reveal any relevant findings. Family history revealed that the patient's mother had similar complaints of mobility, pus discharge, and spontaneous exfoliation of some teeth following which she consulted a dentist and underwent total extraction by the age of 40. The patient was a nonsmoker, and there was no history of use of any other forms of tobacco.

Extraoral examination revealed bilateral submandibular lymph node enlargement, which was firm, mobile, and nontender.

All teeth were present except for 46, 26, and 41. Tooth 46 was extracted due to caries and 41 was extracted due to mobility. Tooth 26 was grossly decayed with just root stump present. The oral hygiene status of the patient was fair with moderate deposits of calculus and plaque.

Intraoral examination revealed a normal color of gingiva except in the labial aspect of 31, 32, and 33 where the marginal gingiva was slightly reddish. Gingival margins were rounded, and exudation was present in relation to labial aspects of mandibular anterior teeth and maxillary central incisors. There was generalized bleeding on probing and recession in relation to most of the teeth, especially more in maxillary central incisors and mandibular anterior teeth.

**Figure 10:**
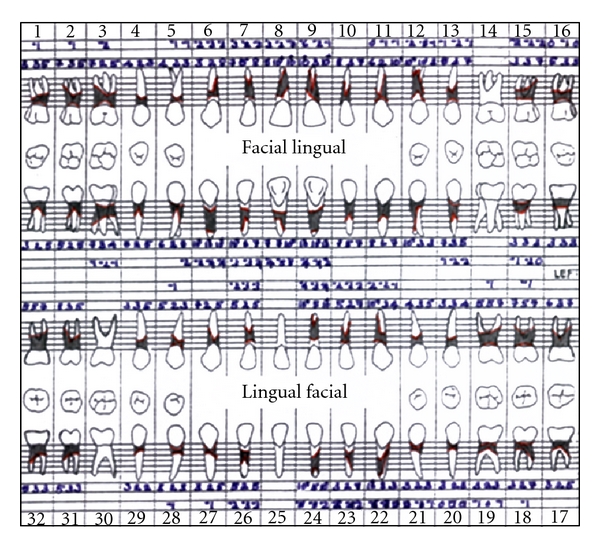
Pretreatment periodontal charting shows deep pockets with generalized clinical attachment loss.

 There was grade I mobility of 15 and 22 and grade II mobility of 11, 12, 21, 31, 32, 33 and 42. Proximal contacts were lost between maxillary and mandibular anterior teeth with pathologic migration of 11, 21, 31, 32, and 42 and extrusion of 31. Grade II furcation involvement was present with molars and maxillary first premolars. A full mouth periodontal examination revealed generalized deep periodontal pockets and severe generalized clinical attachment loss (Figure 10).

Severe periodontal destruction was evident with more than 10 mm of clinical attachment loss at multiple sites especially in the incisor and canine regions.

**Figure 11:**
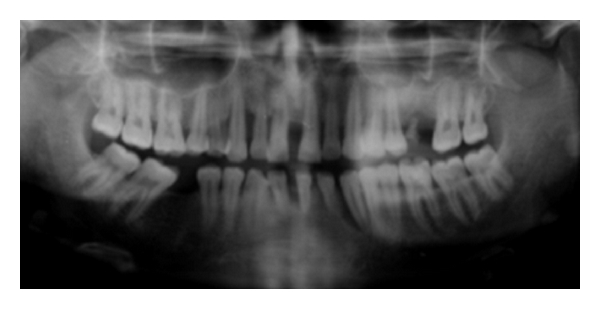
Orthopantomogram showing generalized bone loss with advanced vertical defect in 13 and 33 regions.

 OPG and IOPA X-rays revealed a generalized distribution of periodontal bone loss especially severe in the incisor and canine regions with the molars and premolars affected to a lesser degree (Figure 11). There was predominantly vertical bone loss in the canine and incisor regions. Routine blood investigations were within normal limits.

A diagnosis of generalized aggressive periodontitis was made according to the established criteria (American Academy of Periodontology, 1999).

### 6.4. Management

Supragingival scaling was performed, and the patient was educated in oral hygiene maintenance. The patient was advised to follow a modified Stillman technique of brushing since the patient had root exposure and hypersensitivity and also advised to use interdental brushes and dental floss for optimal plaque control. The patient was prescribed topical antimicrobial agents (metronidazole gel) along with chlorhexidine mouthwash for 2 weeks. A combination systemic antibiotic therapy of amoxicillin and metronidazole [24] was initiated, and a desensitizing agent was prescribed. A recall visit after 2 weeks showed reduction in inflammation and percentage of sites showing bleeding on probing. Exudation was persistent in relation to 11 and 33 regions. A subgingival scaling and root planing was performed following which a povidone iodine 5% irrigation was performed. A nonsustained professionally delivered local drug delivery with metronidazole gel was injected subgingivally at sites 33 and 11, following which a periodontal dressing was given at the site. The procedure was performed every 3 days for the next 2 weeks. Evaluation after 3 weeks showed complete absence of bleeding on probing, exudation, and significant reduction in probing pocket depth. The patient was put on maintenance therapy during which he continued with the topical antimicrobial agents and desensitizing agents and was evaluated for surgical therapy.

**Figure 12:**
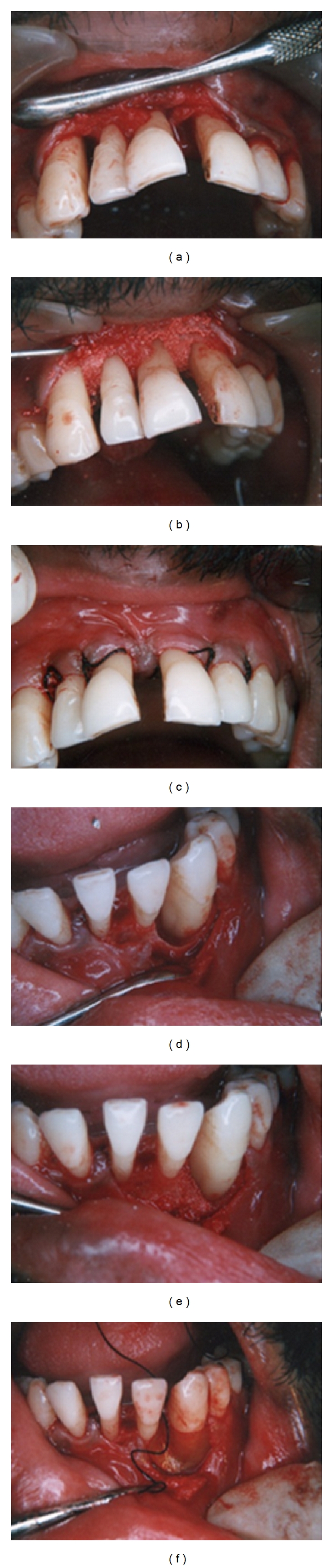
(a)–(c) Flap surgery in conjunction with bone grafting (HAP) and papilla preservation flap between 11 and 21. (d)–(f) Deep circumferential defect treated with bone grafting in conjunction with guided tissue regeneration.

A full-mouth flap surgery with bone grafts (synthetic hydroxyapatite (HAP)), where indicated, was performed sextantwise at intervals of two weeks. In addition, the defect at site 33 was treated with guided tissue regeneration (GTR) with bioresorbable collagen membrane in conjunction with synthetic bone graft (HAP) (Figures 12(a)–12(f)).

**Figure 13:**
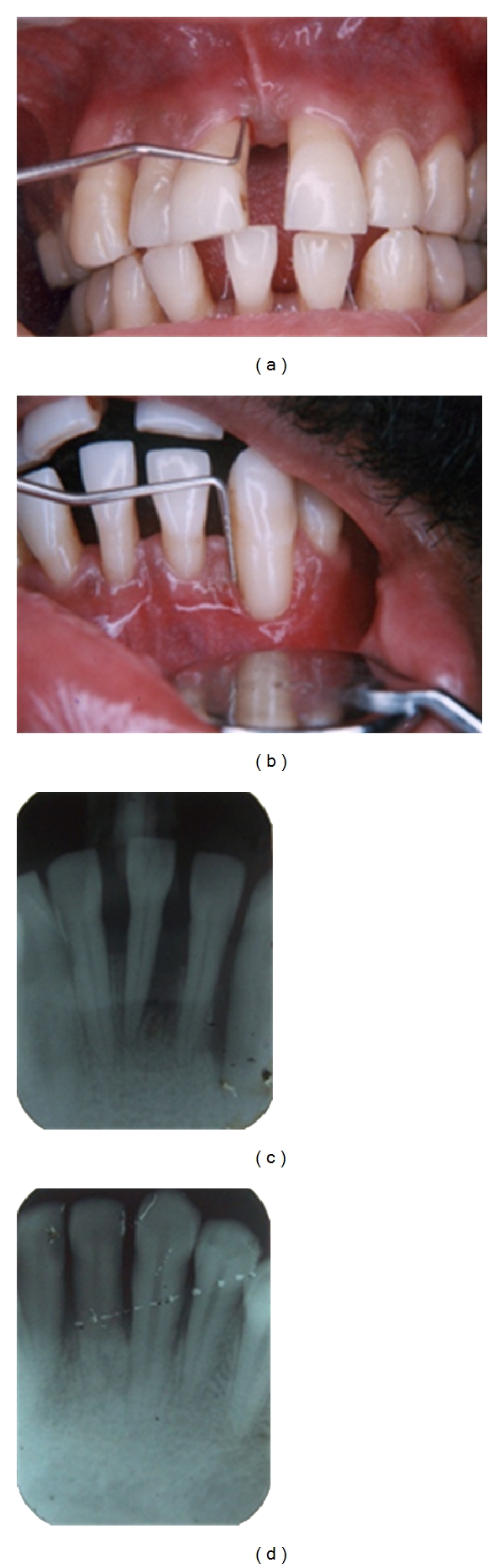
(a) and (b) Postoperative photographs showing probing depths within normal limits with excellent gingival condition; however, there was slight increase in recession after healing. (c) and (d) Comparison of preoperative and 1 year postoperative radiographs shows good bone fill at the defect in relation to 33.

Postoperative clinical evaluation showed excellent gingival condition with reduction in probing depths to normal levels (Figures 13(a) and 13(b)). Radiographs showed bone fill in the region where bone grafts alone or in conjunction with GTR were used (Figures 13(c) and 13(d)). Regular recall appointments were given for maintenance therapy during which the treatment results were well maintained. However, there was a slight increase in recession due to shrinkage of gingiva on healing and hypersensitivity after the surgery which gradually subsided on regular use of desensitizing agents and fluoride mouthwashes.

## 7. Discussion

The key to successful treatment is early diagnosis. Early diagnosis helps in prevention of progression of the disease thus avoiding the possibility of advanced tissue destruction and alveolar bone loss. The earlier the diagnosis is the better the prognosis of the dentition will be. Furthermore since it has a tendency for familial aggregation, it is important to do a periodontal examination of siblings and other close blood relatives of the patient which helps in early diagnosis of the disease in the family members. Management of GAgP patients essentially consists of a nonsurgical phase, surgical therapy an interdisciplinary therapy and a lifelong supportive periodontal therapy.

### 7.1. Nonsurgical/Etiotropic Phase of Therapy

Nonsurgical therapy remains the first line of antimicrobial therapy in GAgP. Early stages of the disease with mild to moderate periodontal and bone destruction may be managed entirely by nonsurgical therapy with systemic antibiotics as an adjuvant to mechanical therapy.

 Therapy should start with attempts at controlling or eliminating the etiologic agents and modifiable risk factors for the disease. The disease has a strong genetic predisposition. The host response of the patient or the susceptible individual to pathogenic bacteria in the dental plaque plays a vital role in the pathogenesis and expression of the disease, and this host response is genetically determined and is an unmodifiable risk factor is at present by the current treatment measures [26]. However, since the expression of the disease in susceptible individuals is also influenced by microbial and environmental risk factors, the disease can be successfully kept under control by controlling the microbial and environmental factors. This underlies the importance of optimal plaque control both by personally employed methods used by the patient himself and professionally employed plaque control measures by the dental team to the patient. Even a minimal amount of plaque is enough to elicit untoward host response in those patients susceptible to the disease, and a reduced resistance to the invasion of subgingival plaque can be compensated for by a correspondingly strong emphasis on total plaque control [27].

Mechanical plaque control can be successfully achieved by educating and motivating the patient if needed with the aid of disclosing solutions regarding the need for optimal plaque control, demonstration of brushing techniques (modified Bass technique for patients without gingival recession and modified Stillman technique in patients with hypersensitivity and generalized recession), and use of interdental cleansing aids like dental floss and interdental brushes where indicated. This behavioral modification from the patient needs a positive reinforcement and encouragement from the dental team. Regular recall appointments to monitor the efficacy of the patient's plaque control measures are essential.

 Chemical plaque control agents like chlorhexidine 0.12% or 0.2% mouthwashes, and 1% povidone iodine can be advised for further plaque control as an adjunct to the patient's mechanical plaque control measures [28]. Amine fluoride and stannous fluoride mouth rinses and tooth pastes as an adjunct to mechanical oral hygiene procedures in GAgP patients were found to be effective in controlling supragingival plaque accumulations in aggressive periodontitis [29, 30]. Additionally use of fluoride mouthwashes is advised to help in remineralization of the exposed root surfaces, and for patients complaining of hypersensitivity, use of desensitizing toothpastes and mouthwashes is mandatory.

Smoking has been well documented as a significant risk factor for aggressive periodontitis with GAgP patients who smoke having more affected teeth and more loss of clinical attachment than nonsmoking patients with GAgP [31]. Furthermore the response to periodontal therapy, both nonsurgical and surgical, regenerative therapy, and implant therapy is less than in nonsmokers, but former smokers respond similar to nonsmokers. This underlies the therapeutic effect of smoking cessation and cessation of other forms of tobacco, and patients should be advised of the benefits of smoking cessation and the potential risks of smoking in worsening their periodontal condition, and if needed expert counseling for cessation of the habit should be sought [32–36].

#### 7.1.1. Mechanical Antimicrobial Therapy

Scaling and root planing (SRP) which eliminates the microbial bacterial load from the periodontal pockets and removes the local etiologic factors is performed either as a quadrant-wise SRP at 2-week interval or as a full mouth scaling and root planning completed on the same day. However, both modalities have been found to be efficacious with significant improvement in clinical parameters, and the clinician should select the treatment modality based on the practical considerations related to the patient preference and clinical workload [37].

Another approach to mechanical antimicrobial therapy is a one-stage full mouth disinfection therapy devised by Quirynen et al., which was found to result in an improved clinical outcome and microbial improvement in early onset periodontitis compared to quadrant-wise SRP [38, 39]. Full-mouth disinfection therapy includes full-mouth debridement (scaling and root planning, brushing of the tongue with 1% chlorhexidine for 1 minute, rinsing of the mouth with a 0.2% chlorhexidine solution for 2 minutes, and irrigation of periodontal pockets with 1% chlorhexidine solution), completed in 2 appointments within a 24-hour period [40].

#### 7.1.2. Photodynamic Therapy and Laser Irradiation

These have been tried as adjuncts to mechanical therapy to inhibit the pathogenic bacteria in periodontal pockets [41–44].

Photodynamic therapy (PDT) is a noninvasive photochemical approach for infection control which combines the application of a nontoxic chemical agent or photosensitizer with low-level light energy and has shown clinical evidence of efficient eradication of periodontal bacteria from subgingival sites [41]. This novel therapeutic approach of antimicrobial therapy seems promising and is getting attention recently either as a monotherapy or as an adjunct to SRP in the nonsurgical treatment of aggressive periodontitis. Both PDT and SRP have been shown to have similar clinical results in the nonsurgical treatment of aggressive periodontitis [42, 43].

Laser irradiation of subgingival sites to eradicate periodontopathic microorganisms is also being considered in the nonsurgical therapy of periodontitis patients. Diode laser treatment has shown a superior clinical and microbiological effect when used along with SRP, compared to SRP alone or laser therapy alone in aggressive periodontitis patients [44].

A regular recall visit preferably at one-week intervals should be performed especially at the initial stages of the treatment to monitor the efficiency of the patient's plaque control measures and to assess the response of the patient towards nonsurgical therapy.

#### 7.1.3. Chemical Antimicrobial Therapy in the Management of GAgP

**Role of Systemic Antibiotic Therapy in GAgP**

Systemic antibiotics are indicated in aggressive periodontitis since the pathogenic bacteria like *Aggregatibacter actinomycetem-comitans* and *Porphyromonas gingivalis* have been found to be tissue invasive and mechanical therapy is insufficient to eliminate the bacteria from these sites [58, 59]. Systemically administered antibiotics with or without scaling and root planning and/or surgery provided greater clinical improvement in attachment level change compared to similar periodontal therapy without antibiotics [45]. Earlier tetracyclines were used extensively for this purpose since systemic tetracycline was found to be a useful adjunct to mechanical periodontal therapy in patients with aggressive periodontitis [46–48], but the concern for tetracycline resistance has shifted the focus to the use of other antibiotics both as combination therapy or serial antibiotic therapy [49].

The preferred combination antibiotic therapy at present for treatment of GAgP is 250 mg of amoxicillin thrice daily along with metronidazole 250 mg twice daily for 8 days [24, 49]. It is one of the most evaluated drug combinations in GAgP, and there is ample evidence now to show that Amoxycillin-Metronidazole combination as an adjunctive treatment in GAgP at initial therapy significantly improves the results and hence should be preferred over other antibiotic regimens as the first-line treatment (Table 1) [50–55].

The usefulness of microbial testing may be limited because of the variability of test reports between different labs and the mixed flora, and hence an empiric use of antibiotics like the above-mentioned combination may be more clinically sound and cost-effective than bacterial identification and antibiotic-sensitivity testing in the treatment of aggressive periodontitis [49].

Single-agent therapy with Doxycycline [53, 55], azithromycin [56], metronidazole [53, 57], and clindamycin [57] is effective when used adjunctively to nonsurgical procedure of SRP in AgP patients. The criteria for selection of antibiotics are not clear in AgP; the choice depends on the case, disease-related factors and patient-related factors like compliance, allergies, and potential side effects.

#### 7.1.4. Local Drug Delivery of Antimicrobial Agents

Topical application of antimicrobial agents and local drug delivery is also a treatment option especially if there are localized areas of exudation and deep pockets not responding adequately to mechanical and systemic antibiotic therapy. Local drug delivery delivers the drugs at high concentrations at the site of infection when compared to systemic antibiotic therapy. Furthermore, this is an option in patients where there is intolerance to systemic administration of the antibiotic.

 Several local anti-infective agents combined with SRP appear to provide additional benefits in PD reduction and CAL gain compared to SRP alone. Over the past 20 years, locally delivered, anti-infective pharmacological agents, most recently employing sustained-release vehicles, have been introduced to achieve this goal [60].

Though there is more evidence on its application in chronic periodontitis, till future researches are available; the same agents can be employed in aggressive periodontitis patients as well empirically. Adjunctive use of LDD agents like controlled release biodegradable chlorhexidine gluconate chip [61, 62], tetracycline fibers [63, 64], and minocycline-Hcl gel [65] has been tried in aggressive periodontitis with superior clinical outcomes. The decision to use local anti-infective adjunctive therapy remains a matter of individual clinical judgment, the phase of treatment, and the patient's status and preferences.

An evaluation of the response to nonsurgical treatment is done 2-3 weeks after treatment during which the gingival and periodontal status of the patient will be reevaluated and compared with the pretreatment values to assess the response to therapy and to assess the areas which need surgical therapy. Sites with persisting pockets >5 mm depth, vertical bone defects which need regenerative therapy, difficult to instrument areas like furcation involvement, and areas which need recontouring or resective osteoplasty are indications for surgery.

### 7.2. Surgical Therapy

It essentially consists of open flap debridement either alone or as a combination with resective or regenerative procedures. The main aim of a flap procedure is to get access and visibility to root and furcation areas so that a thorough instrumentation and debridement can be performed. Flap techniques like modified Widman flap [25], modified flap operation/Kirkland flap (sulcular incision flap) [66] achieve this aim without eliminating the pockets. A resective flap procedure like undisplaced flap [67] will eliminate the pockets as well but compromise the esthetics and function of the dentition by root exposure and resultant hypersensitivity and hence is not preferred usually when compared to modified Widman flap or sulcular incision flap.

Laser-assisted surgery (Nd: YAG laser) is suggested as a valid alternative to conventional scalpel surgical therapy, in individuals at increased surgical risk like in coagulation and platelet function disorders [68].

#### 7.2.1. Regenerative Surgical Therapy

Regeneration of the periodontal supporting structures lost due to periodontal disease so that the form and function of the periodontium is reestablished has been an elusive or difficult-to-achieve goal for periodontal therapists.

Various modalities are being employed for periodontal regeneration which includes use of bone replacement grafts, barrier membranes or guided tissue regeneration (GTR), biologic modifiers like growth and differentiation factors (GDF), and extracellular matrix proteins like enamel matrix proteins (EMD) or use of a combination of the above techniques and materials which has been extensively reviewed elsewhere [69].

A sulcular incision flap or papilla preservation flap will be the ideal technique to minimize recession in the anterior regions due to esthetic reasons, and modified Widman flap or conventional/sulcular incision flap will be the technique of choice in the posterior regions when opting for bone grafting and another regenerative therapy. A papilla preservation flap is preferred for bone grafting when there is spacing between the teeth to obtain maximum coverage of the graft material at the interdental region and to prevent shrinkage of papilla on healing [67]. Biomodification of the root surface (Root conditioning) with citric acid, tetracycline, or fibronectin is preferable when performing bone grafting or GTR for better clinical results [69].

#### 7.2.2. Bone Replacement Grafts

Bone grafting is indicated in vertical defects, and the success of the procedure depends on the type of defect. Three-walled or intrabony defect is the ideal defect for bone grafts and has a better success rate compared to a two-walled and one-walled defect. The type of bone graft which gives the maximum benefit with minimum tissue reaction is autograft [70], but there are limitations of obtaining it in large quantities as is needed in most cases of generalized aggressive periodontitis. A more feasible option is to use commercially available bone grafts, which are allograft, xenograft, or alloplastic materials.

Allografts used for periodontal grafts include mineralized freeze-dried bone allografts (FDBAs) which are osteoconductive, and decalcified freeze-dried bone allografts (DFDBAs) which are osteoinductive. Decalcification of the graft exposes the complex bone morphogenic proteins (BMPs) from its matrix which can induce osteoblastic proliferation in the recipient site. DFDBA, because of its osteoinductive property, has shown to have better results than the alloplastic materials which are osteoconductive [71]. Allogeneic freeze-dried bone (FDBA) mixed with tetracycline powder along with systemic tetracycline has demonstrated a better clinical outcome in treatment of juvenile periodontitis [72].

Xenografts used are either bovine derived or coral derived. An osteoconductive bovine-derived anorganic bone, Bio-Oss, has been successfully used in periodontal defects with resulting bone regeneration and new attachment in these defects [73–75]. Human histologic studies have shown that a combination of Bio-Oss with either purified porcine collagen (Bio-Oss Collagen) [76] or a synthetic cell-binding polypeptide (Pepgen P-15) [77] has the capacity of inducing regeneration of the periodontal attachment apparatus when placed in intrabony defects. Coralline grafts implanted into human periodontal defects have produced better clinical results when compared to nongrafted sites [78].

Synthetic grafts/alloplastic grafts have been considered primarily as defect fillers. The most commonly used among alloplastic graft materials is hydroxyapatite (HAP) which is osteoconductive and has shown to have similar clinical effect to FDBA [79]. Other alloplastic grafts which can be used are beta tricalcium phosphate and bioactive glass [80, 81].

 A synthetic hydroxyapatite/equine type I collagen/chondroitin sulphate biomaterial (Biostite) has been found to show comparable improvements to Bio-Oss in terms of clinical attachment gain, pocket depth reduction, and radiographic bone fill in the treatment of deep intraosseous defects [82].

#### 7.2.3. Guided Tissue Regeneration

Guided tissue regeneration promotes regeneration by acting as a barrier which prevents apical migration of epithelium and exclude gingival connective tissue from the healing wound, thus allowing the pluripotent periodontal ligament cells to populate the site of healing enhancing new cementum and new attachment procedures.

GTR has shown to have a greater effect on probing measures of periodontal treatment than open flap debridement alone, including improved attachment gain, reduced pocket depth, less increase in gingival recession, and more gain in hard tissue probing at reentry surgery [83]. Research has shown that GTR in conjunction with bone grafting has better potential for regeneration compared with either technique alone [74, 84, 85], and this outcome has been confirmed in aggressive periodontitis also with the use of bioresorbable membranes (Bio-Gide) [75, 80].

#### 7.2.4. Biologic Mediators and Extracellular Proteins

A wide array of regenerative materials is being considered for use in periodontitis. Use of biologic mediators like growth factors (insulin-like growth factor (ILGF), platelet-derived growth factor (PDGF)) use of platelet-rich plasma which contains PDGF, extracellular matrix proteins like emdogain, etc. are of promising results. Application of enamel matrix proteins alone [86] or in combination with bone grafts including bioactive glass has shown to result in the successful treatment of intrabony defects in aggressive periodontitis [87].

Beneficial effects of platelet-rich plasma (PRP) in the treatment of periodontal defects have been demonstrated by clinical and radiographic measurements together with reentry results showing marked improvements from baseline with increased stabilization of whole dentition including the hopeless teeth [88, 89].

Various commercially available regenerative materials including bone replacement grafts, GTR membranes, enamel matrix derivatives, are in the market for use in periodontal therapy with varying results, and the choice of the material depends on the dentist's preference and experience with the products helping in clinical judgment of the therapeutic results of individual products and procedures and their cost-benefit ratio.

### 7.3. Role of Maintenance Therapy in Management of Aggressive Periodontitis

The importance of supportive periodontal therapy has to be stressed in management of aggressive periodontitis. Regular SPT was found to be effective in maintaining clinical and microbiological improvements attained after active periodontal therapy in early onset periodontitis [90].

The maintenance therapy starts soon after the phase I therapy or nonsurgical therapy and should be continued throughout the lifetime of the patient. Or in other words, “*maintenance therapy never ends*” for a GAgP patient. In order to maintain the optimal results got by surgery and to prevent the recurrence of the disease, a lifelong maintenance therapy is mandatory because of the strong genetic susceptibility of the individual to the disease.

The frequency of the recall visits depends on the response of the individual to treatment and presence of other risk factors like environmental factors but generally will be more frequent than that in chronic periodontitis or in localized aggressive periodontitis. Any site which shows signs of recurrence of the disease like bleeding on probing which is considered as the first clinical sign of inflammation should be treated vigorously and monitored for resolution of the signs.

### 7.4. Interdisciplinary Approach for Management of Resultant Esthetic, Functional, and Psychologic Problems in GAgP

A comprehensive management for total rehabilitation of the GAgP patients not only involves control of infection and arrest of progression and/or regenerative therapy by the periodontist but also incorporates a multidisciplinary approach to attend the esthetic, functional, and psychologic problems faced by the patient.

An orthodontic therapy with concomitant periodontal monitoring and prosthetic rehabilitation, if possible with the use of implants and psychologic counseling, may be needed for patients with advanced forms of the disease.

#### 7.4.1. Combined Periodontal-Orthodontic Therapy

Cosmetic concerns in young aggressive periodontitis patients will be high since the disease can result in flaring, protrusion, pathologic migration, and even extrusion of the anterior teeth. Malocclusion, pathologic migration and potential occlusal traumatism which can cause secondary trauma from occlusion can be corrected by orthodontic therapy in GAgP patients already stabilized by periodontal therapy [91–94]. Orthodontic treatment can be commenced once attachment gain and bone stability is achieved after periodontal therapy but is generally advised to postpone till 3 months to 1 year after active periodontal therapy. A combined periodontal and orthodontic treatment demands a detailed evaluation in both specialties, particularly when the periodontium is reduced. Periodontal evaluations are scheduled concomitantly with orthodontic appointments to monitor the periodontal stability as the tooth movement occurs.

#### 7.4.2. Prosthodontic Rehabilitation, Implant Therapy, and Implant Supported Prosthesis

Gingival recession with loss of interdental papilla especially in the anterior teeth is unaesthetic especially when the patient smiles and the feasibility of root coverage periodontal plastic surgery will be limited in generalized aggressive periodontitis because of the large number of teeth involved and the advanced interdental bone loss. A porcelain, resin, silicone, or copolyamide removable gingival prosthesis (gum veneer/gingival mask) can be fabricated to mask the recession and improve the appearance of the anterior teeth [95]. The restoration of the teeth lost due to periodontitis should be done with fixed or removable prosthesis depending on the bone support of the remaining teeth.

Contradictory to the earlier concept that implants are not a feasible option in GAgP patients, the use of implants and implant-supported prosthesis to restore the lost teeth is increasingly considered as a treatment option in well-maintained GAgP patients even though the risk of bone loss and attachment loss around implants is higher than that in chronic periodontitis patients or periodontally healthy individuals, with researches showing good survival of implants over a 10-year period [96]. Several reports are there which have successfully used osseointegrated implants in oral rehabilitation of partially edentulous patients treated for GAgP [97–99].

#### 7.4.3. Psychotherapy

Perhaps the least recognized and the most underestimated aspect in the total rehabilitation of a patient with GAgP presenting with multiple tooth loss and/or advanced periodontal destruction necessitating extraction of multiple teeth is the need for psychological counseling and psychotherapy. It aims at attending the psychologic effect and potential mental depression following tooth loss due to rapid periodontal destruction which provides the patient with relatively less time to cope with the situation. The emotional effects of tooth loss are devastating for some patients and have a dramatic impact on their life, and they take longer time to come to terms with the tooth loss [100]. Preparing the patients with advanced disease having multiple teeth with hopeless prognosis emotionally for extraction also has to be dealt with carefully by the dentist, if needed using multiple appointments, and the extent of the impact that bad news, such as having to lose teeth, has on an individual is most often dependent on the way in which the information is communicated [101]. Depression, anxiety and social withdrawal are seen in patients with tooth loss, and resulting compromised esthetics can be helped with therapy, relaxation techniques, and, in some cases, antidepressants. Any of the above symptoms should be addressed with a qualified psychotherapist to improve the quality of life.

Psychotherapy has to be started immediately following the first appointment and should be continued concomitantly for total rehabilitation of the patient for a variable duration depending upon the psychologic status of the individual patient. In addition, stress reduction protocols may help in management of the disease as such in the view of the recent suggestions of the proposed mechanisms by which stress can contribute to the onset, exacerbation and maintenance of the periodontal disease [102]. A recent study reported that psychotherapy offered at 3 levels (individual, group, and conjoint family psychotherapy) to GAgP patients gave positive psychologic effects that restored their ability to socialize in their environment contributing to their positive experience in life [103]. The above facts suggest that psychotherapy be incorporated for the future protocols for treatment of GAgP patients suffering from emotional effects of tooth loss.

#### 7.4.4. Other Treatment Modalities and Future Trends in Management of Aggressive Periodontitis

Host modulation therapy with systemically and locally administered agents is under research for therapy in aggressive periodontitis. Subantibacterial dose of Doxycycline has been approved for use in chronic periodontitis, but its use in aggressive periodontitis has to be confirmed by research. Adjunctive use of locally administered alendronate gel with SRP for host modulation has shown promising results in aggressive periodontitis [104].

 Newer generations of regenerative materials and advances in tissue engineering for regeneration and genetic engineering to modify the genetic risk factors seem to be really promising in the future. With further understanding of the genetic risk factors, a futuristic application of genetic screening tests will be in identifying the susceptible individuals and instituting the preventive measures to keep the gene expression and thus the disease under control [105, 106].

#### 7.4.5. A Suggested Protocol for the Comprehensive and Total Rehabilitation of GAgP Patients with the Current Treatment Modalities

**Figure 14:**
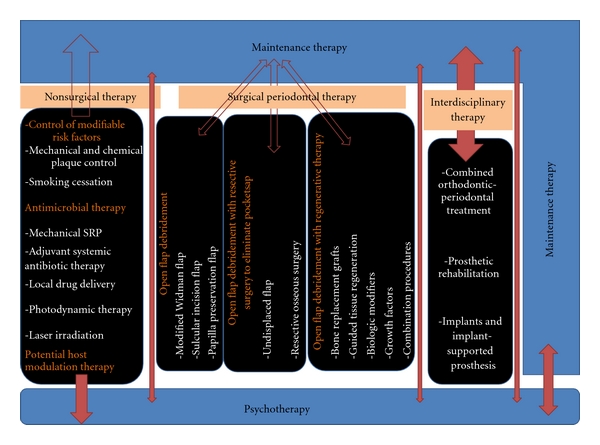
The treatment commences with a nonsurgical phase following which psychotherapy sessions are started for the needy patients, and patients are put on maintenance therapy/supportive periodontal therapy thereafter. While the patient is on maintenance therapy, one or more of the 3 modalities of surgical periodontal therapy can be performed depending upon the indication and once periodontium has been stabilized, an interdisciplinary treatment for the restoration of lost teeth and correction of cosmetic problems is completed following which the patient continues for a “lifelong” maintenance therapy.

For more details see Figure 14.

## 8. Conclusion

Even though the prevalence of aggressive periodontitis is much lower than chronic periodontitis, the management of aggressive periodontitis is more challenging compared to that of chronic periodontitis because of its strong genetic predisposition as an unmodifiable risk factor. Researchers are going on employing the potential several novel technologies in regenerating the lost periodontium including tissue engineering and genetic engineering.

The key to successful management at present lies in early diagnosis of the disease and rigorous treatment employing the different treatment modalities mentioned in the paper along with systemic antibiotic therapy followed by meticulous lifelong maintenance therapy. With the current treatment modalities, successful long-term maintenance of the dentition in a healthy and functional state can be achieved. A comprehensive periodontal treatment consisting of mechanical/surgical and systemic antimicrobial therapy is found to be an appropriate treatment regimen for long-term stabilization of periodontal health with arrest of periodontal disease progression in 95% of the initially compromised lesions [107].

Further understanding of the etiology, risk factors, pathogenesis, and host immune response in aggressive periodontitis along with advances in regenerative concepts, tissue engineering, and gene therapy is needed for formulating better management protocols in the treatment of generalized aggressive periodontitis.

## Figures and Tables

**Table 1:** The list of antibiotic regimens with evidence of superior clinical outcome when used as an adjuvant to SRP in GAgP [24, 45–57].

Mode of therapy	Antibiotics used	Usual recommended dosage
Combination therapy	Metronidazole + Amoxicillin	250 mg of each thrice daily for 8 days
	Metronidazole + Ciprofloxacin	500 mg of each twice daily for 8 days

Single-Agent therapy	Doxycycline or Minocycline	100–200 mg once daily for 21 days
	Metronidazole	500 mg thrice daily for 8 days
	Tetracycline	250 mg 4 times daily for 1 week
	Azithromycin	500 mg once daily for 3 days
	Clindamycin	300 mg thrice daily for 10 days
